# Eating Habits and Disease Risk Factors

**DOI:** 10.3390/nu14153143

**Published:** 2022-07-30

**Authors:** Katarzyna Eufemia Przybyłowicz, Anna Danielewicz

**Affiliations:** Department of Human Nutrition, Faculty of Food Sciences, University of Warmia and Mazury in Olsztyn, ul. Słoneczna 45F, 10-718 Olsztyn, Poland

Diet plays an inevitable role in human health and disease prevention. However, personal dietary choices involve not only what food and nutrients are consumed but also why, how, and under what circumstances. Focus on eating habits allows us to observe the complexity of socio-behavioural, economic, environmental and cultural determinants of the human diet. Individual eating habits, due to their longitudinal character, represent an important contributor to disease. On the other hand, eating habits can be modified, and therefore are a promising target for lifestyle interventions, which can influence future health. Moreover, as many factors influence eating habits, it is vital to understand how changes in those factors can affect the human diet.

Considering the development of eating habits, it is essential to pay attention to the behaviours of children and adolescents in relation to nutrition to avoid developing and repeating inappropriate patterns in adulthood. In one of the papers presented [1], adolescents’ behavioural eating patterns were identified as “Prudent Ones” when they had knowledge about healthy eating and followed the recommendation, “Inconsequent” when follow recommendation partially, and “Rebels” when adolescents follow an unhealthy diet and eating behaviours. In this work, it was found that those more likely to follow healthy eating patterns and behaviours were students of female sex, with no siblings, better nutrition knowledge, and greater physical activity in leisure time. The adherence to the “Rebels” was observed in those of male sex, students with families larger than four people, whose mothers were in primary education and who had insufficient nutrition knowledge. However, none of the patterns were associated with BMI [1].

Emotional eating is a disordered eating behaviour that often leads to inappropriate food choices. The Bui et al. [2] demonstrated that undertaking other activities, including binge drinking, smoking, and being sedentary, were associated with the consumption of unhealthy foods. Meanwhile, reading the nutrition labels was the only factor that was associated with decreasing frequent unhealthy food consumption.

Eating speed is a behaviour that is often overlooked when evaluating overall eating habits. It can significantly affect the speed of reaching a feeling of satiety and the amount of food consumed, which can lead to further health implications. Garcidueñas-Fimbres [3] in her narrative review summarizes that faster eating speed may lead to increased risk of developing adiposity, metabolic syndrome, or its components, whereas higher eating frequency could related to diet quality improvement, lower adiposity, and a lower risk of metabolic syndrome [3].

Over recent decades, there has been a growing trend to promote healthy eating, largely based on unprocessed, plant-based foods and organic and ecological products. On the other hand, the industry is providing us with many new products derived by new methods or using new food sources. The disorders related to the mentioned trends are orthorexia nervosa, which can be shortly described as a pathological obsession with eating only healthy food and excessive worrying regarding healthy dietary intake [4], and food neophobia, which is related to the reluctance to eat or the avoidance of new food [5]. A study of Brytek-Matera et al. [4] shows that the prevalence of orthorexia nervosa may be different in different regions of the world. In Lebanon, which is representative of the non-Western world, adults’ orthorexia prevalence was higher than in Polish ones, with no correlation for age in both samples. Furthermore, higher orthorexia tendencies in Lebanese adults were associated with higher BMI and those whose marital status was single, whereas no associations were observed in the Polish sample [4]. In turn, Jeżewska-Zychowicz et al. [5] indicates that food neophobics were older, had a lower level of education, had higher BMI, were more likely to consume vegetables, fruit, meat, and meat products, and rarely consumed functional and convenience food, sweets, and sweetened beverages. Moreover, food neopobics chose healthy and tasteless food products and did not read price and shelf-life information on food labels compared to food neophilic or neutral adults [5].

We must not forget that the recent global situation arising from the COVID-19 pandemic has also not been without implication on dietary habits and the motives that guided the choice of particular products. A survey of Croats [6] showed that during the pandemic, health was ranked as the number one most important motive for food choice, whereas before, sensory appeal guided the selection. Additionally, researchers found a difference in food choice motives between gender.

The evaluation of eating habits and behaviours is essential for taking appropriate further steps in both research and clinical practice, which will be adjusted to the specificity of different populations. The validation findings of Arhire et al. [7] will enable us to use Dutch Eating Behaviour Questionnaire among Romanian adults, whereas the validation of the Spanish version of the Eating Habits Questionnaire demonstrated strong internal consistency and suitability for assessing orthorexia nervosa in Spanish young adults [8].

An important part of diminishing the risk of diseases and supporting their treatment is the use of proper diet. A nutritional matrix enriched with appropriate products and bioactive components can prevent the onset of diseases and contribute to prolonging life and ensuring its good quality.

In recent years, special attention has been paid to the group of methylxanthines that includes derivatives such as caffeine, theobromine, theophylline, pentoxifylline, or propentofylline. Given their anti-inflammatory and antioxidant properties, its association with the risk of the onset of various chronic non-communicable diseases (NCDs) is being studied. Reviewed trials [9] provide beneficial, protective effect on pathological process leading to neurodegenerative disease. However, the usual daily intake is not sufficient to trigger the effects observed in the studies, and the use of higher pharmaceutical doses is needed. Nevertheless, it is important to be aware of the unintended side effects of taking an increased intake [9]. On the other hand, a study in a sample of Saudi adults with type II diabetes showed that total caffeine intake from various sources was not associated with markers of glucose control and cardiovascular risk [10].

The NCD that burdens an increased number of population is cancer. Numerous studies provide data on the positive effect of food and its compounds in its prevention. In the study of Wajszczyk et al. [11], the consumption of dairy products had a bi-directional association with breast cancer risk in women, which depended on product type and menopause status. For colorectal cancer, dietary habits can indirectly modulate the risk of its occurrence, through the stimulation of microbiota for the production of short-chain fatty acids (SCFA), notably phytate and butyrate. Markiewicz et al. [12] mentioned that SCFA in combination enhanced their pro-apoptotic effect in cancer cells, while suppressed pro-proliferative action and activated pro-survival pathway was observed for phytate in the healthy cells.

Small dense low-density lipoprotein (sdLDL) due to their atherogenicity is associated with an increased risk for ischaemic heart disease. The study [13] showed that the change of a low saturated fatty acid diet with an increased amount of protein, at the expense of carbohydrates, had beneficial effects on LDL particle distribution with decreasing sdLDL in mildly hypercholesterolemic subjects. Additionally, the effect was higher in subjects with increased insulin resistance [13].

In addition to the occurrence of diseases themselves, prevention is also important. Intrauterine metabolic programming of the foetus through the mother’s diet can determine the occurrence of diseases in the offspring with adult life. Mazurkiewicz and Bronkowska [14] showed that the consumption of sweet and salty snacks, fruits, and fruit and vegetable juices determined newborn birth weight, while the consumption of sweet and salty snacks and grain products was related to weight gain during pregnancy. Moreover, it was shown that women aged 35 and younger consumed more animal fats than those over 35 years of age. However, no correlation was found between levels of insulin and IGF-1 and mothers and the birth weight of the newborn [14]. It is also supposed that disordered eating behaviours may impact newborn birth characteristics. The study of Sámano et al. [15] supported a relationship with newborn health outcomes; they found that stronger restrictive, compensatory, and binge–purge eating disorders may lead to an increased gestation weight gain in adolescent mothers [15], which can indirectly modulate newborns’ health parameters in their development.

It is worth noticing that changes in eating habits of the whole population, which are observed over the years, may affect their health. The review of Wulan et al. [16] highlighted the westernization of the diet and decrease in physical activity among the male population of South Asia, which led to the rapid development of overweight and obesity, diabetes, and metabolic syndrome. Authors indicate that it could be an effect of differences in energy expenditure and substrate utilization, as well as insufficient molecular adaptation to overfeeding with a high-fat diet in comparison to the Caucasian population [16].

In conclusion, the mentioned works show how, in addition to simply assessing food intake, it is important to assess other determinants of changes in eating habits. Their assessment can also help in the proper selection of nutritional–behavioural interventions and the planning of targeted primary prevention campaigns. Along with the proven effects of specific dietary strategies that take into account the beneficial effects of individual bioactive compounds, we can design dietary interventions tailored to the needs of different populations, both maintain the health of healthy people and to aid the process of treatment for those who are suffering from disease.
